# Relationships between training dose and record power outputs in professional road cyclists: insights and threats to validity

**DOI:** 10.5114/biolsport.2023.114284

**Published:** 2022-07-21

**Authors:** Gabriele Gallo, Andrea Bosio, Marco Martin, Andrea Morelli, Matteo Azzolini, Luca Guercilena, Josu Larrazabal, Ermanno Rampinini

**Affiliations:** 1 Mapei Sport Research Centre, Olgiate Olona, Italy; 2 Centro Polifunzionale di Scienze Motorie, University of Genoa, Genoa, Italy; 3 TrekSegafredo, Deinze, Belgium

**Keywords:** Endurance sports, Training data, Cycling races, Race data, Grand tours, One-day races

## Abstract

Testing short-term (4–8 weeks) correlations between training dose and performance in professional road cyclists can help improve their training and performance. Multilevel mixed-modeling was used to correlate training dose (Time, Edwards’ Trimp-eTRIMP, Training Stress Score-TSS, time spent in power output zones-Z1, Z2, Z3, Polarization Index-PI) and Record Power Output (RPO) over 1 minute (RPO1), 5 (RPO5), 20 (RPO20), and 40 minutes (RPO40) across four different time periods: training dose of the previous month with RPOs of the subsequent month (Monthly-analysis); training dose of the 8 weeks preceding All, Grand tours, One-day races with RPOs of these races. In Monthly-analysis, small positive relationships between all the training dose parameters, except for PI, and RPO1, RPO5, RPO20, RPO40 were found (p ≤ 0.001). In Grand tours analysis, Z3 showed a positive association with RPO40 (r: 0.45; p = 0.007, moderate) and was positively related to RPO1 and RPO5 (r between 0.32 and 0.34; p = 0.053–0.059, moderate). PI was small positively related to RPO1 (r = 0.29, p = 0.076, small). In One-day races analysis, eTRIMP was positively related to RPO5 (r = 0.30, p = 0.035, moderate), Z1 negatively related to RPO40 (r = -0.31, p = 0.031, moderate), PI positively related to RPO5 (r = 0.24, p = 0.068, small) and Z2 was negatively related to RPO20 (r = -0.29, p = 0.051, small). A certain degree of responsiveness to training dose exists in professional road cyclists. To improve RPOs an appropriate preparation pattern seems to be increasing high intensity training for Grand Tours and fostering high intensity and overall training load (eTRIMP and TSS) in a more polarized-fashion for one-day races. Systematic and precise data collection during training and racing is highly advocated.

## INTRODUCTION

High-level endurance sports require a careful management of training dose with the right balance between training load and recovery to achieve the best performance [1, 2]. Modeling the dose-response relationship between training and performance is crucial because it allows to have greater proactivity (i.e. planning the input based on an expected response) in manipulating training dose, rather than reacting to a response (performance or test) [3].

In endurance sports, the training dose is commonly described by two main concepts: (i) training load (TL), i.e. variables which take into account both volume and intensity of the exercise session [4]; (ii) training intensity distribution (TID), i.e. how intensity is distributed over time during exercise [5]. Training dose could be distinguished as external or internal depending on whether it refers to the objective measure of the work that an athlete completes (i.e.: work, power, distance, velocity) or to the individual psychophysiological response to cope with the external load (i.e.: heart rate, perceived exertion) respectively [1, 6].

Despite evidence suggested that a higher overall training load [7] and a polarized training intensity distribution [8–11] could induce superior training adaptations and performance gains in non-professional endurance athletes, only a few studies have investigated the relationship between training load and performance in competitive athletes [3, 12]. Sanders et al. [3] monitored external and internal training load, measured as Bannister Training Impulse (TRIMP), Edwards TRIMP, Lucia TRIMP, individualized TRIMP, training stress score (TSS), session ratings of perceived exertion (sRPE), in a group of cyclists over 10 weeks during the preparation period. They observed a moderate increase in maximal oxygen consumption and power output at 2 mmol · L^-1^ and small increases in power output at 4 mmol · L^-1^, Wmax, mean absolute (W) and relative power output (W · kg^-1^) during the 8-min maximal power test repeated after the training period. From large to very large positive relationships between both external and internal training load and performance outcomes, measured as percentage changes in power outputs at blood lactate concentration of 2 [mmol · l^-1^] and 4 [mmol · l^-1^] and 8-min maximal power test [3].

On the contrary, Foster et al. [12] did not find relationships between changes in time trial performance and training time or sRPE during a 6-week period in a group of cyclists, runners, and speed skaters. Interestingly, the same authors [12] monitored variations in training load and time trial performance in a small subgroup of athletes for a longer period and suggested the presence of a log curve relationship. Unfortunately, these data were just explorative and observational.

Making an attempt to describe the relationship between training dose and performance in professional road cycling, one of the main problems is to define performance among different races. In fact, in mass-start road cycling races, performance may not be simply identified with the order of arrival or with the time taken to complete the race [13]. In cycling, differently from some other endurance sport (i.e., running and swimming), different races have different distances, altimetric profile (flat, semi-mountainous, mountainous) and tactical dynamics [14]. Furthermore, personal race result is not the primary goal of gregarious cyclists, whose the main assigned task is helping the team leaders obtain the best placement possible at the finish line. Although extensive research has been conducted on the physiology of professional cyclists [15–17] and the exercise intensity and load during different cycling races [18–20], evidence regarding a clear link between training dose and racing performance seems to lack in the literature.

A possible solution could be using, as performance variables, the three main physiological laboratory parameters (maximal oxygen consumption, lactate threshold and efficiency) indicated by Joyner and Coyle as the main determinant factors in endurance sports [21]. However, it has not been clearly established whether physiological laboratory parameters are positively correlated or not with performance in professional mass-start road cycling races and so if they represent or not a valid and absolute performance parameter. Indeed, the few studies that reported a positive correlation between physiological laboratory parameters and performance in professional road cycling only considered time trial races [22]. Moreover, regularly performing lab tests with professional cyclists is very difficult due to the high number of travel and races (especially during the competitive period, from January to October), thus alternative cycling performance parameters would be preferable in order to build a dose-response model with a sufficient frequency of data collection also during the competitive period. In the last years, there has been a rapid increase in the capacity to capture real-time data through portable mechanical power meters [23]. They represent an important tool to monitor an index of physical performance such as power output both in races and daily training sessions. One of the most widely used power meters-derived physical performance indicator is the Record Power Profile [24]. It can be defined as the highest mean recorded power output (RPO) over a given duration) [24]. The commonly considered durations are: 1, 5, 30 and 60 s or 5, 10, 20, 30, 45, 60, 120, 180, and 240 min [24]. Van Erp and colleagues [25] recently suggested that RPOs could be used as physical performance parameters. They showed that RPOs over short durations (< 5 min) were higher for a top-10 result compared to a no top-10 in one team which achieved great success with sprinters [25]. Moreover, Leo and colleagues [26] showed how Record Power Profile combining both classic RPOs and RPOs after a total amount of work (1000–3000 kJ) predicted the final general ranking and UCI points scored during the multistage race Tour of the Alps.

Only two studies have investigated the relationship between training dose and Record Power Profile. Pinot and Grappe [27] reported a significant correlation between average weekly training load (sRPE) and annual increase in RPOs for durations between 5 minutes and 4 hours in a six-year (from the Under 19 to the Professional category) longitudinal case study on a world-class cyclist. Leo and Colleagues [28] reported a significant correlation between changes in training characteristics and changes in the power profile between early- and mid-season, but not between mid- and late-season in Under 23 cyclists. However, to our knowledge, no studies have investigated the relationship between training dose and RPOs within a relative short period (i.e.: 4–8 weeks) in professional cyclists.

Therefore, the aim of this study was to test whether there is a short-term correlation between training load measurements and training intensity distribution with some RPOs over different durations (between 1 and 40 min) in professional road cyclists. Definition of training dose-physical performance relationships may assist to develop training strategies more effectively to improve road cycling performance.

## MATERIALS AND METHODS

### Participants

Forty-six male professional cyclists, members of a World Tour professional cycling team, participated in this study (mean ± SD: 29.6: ± 5.0 yrs, height: 181.0 ± 5.9 cm, body weight: 71.4 ± 7.9 kg). Cyclists participating in the study won some of the most important road cycling competitions during the analyzed period: Olympic Games, World Championship, Giro di Lombardia and stages at Giro d’Italia, Tour de France and Vuelta a España. Annually, they rode an average of 28876 ± 3294 km, raced on average 74 days, and totalized 360 ± 493 UCI points.

### Ethics

The study was approved by an independent review board in accordance with the Declaration of Helsinki for the Human Rights and a written informed consent was signed by all the participants prior to commencing the research as a common procedure for the World Tour professional cycling team they belonged to.

### Data collection

Over a period of 4 consecutive seasons (from the 2016 to the 2019 season), heart rate (HR) and power output (PO) data were daily collected during both training and races, using a HR chest belt and power meters (SRM GmbH, Jülich, Germany or DURACE FC-R9100-P, Shimano, Sakai, Osaka, Japan) that were zeroed before every ride. The accuracy of these power meters was reported in a previous study [29]. For each cyclist, data from one to four seasons were analyzed. Daily data were visually checked by three researchers with pluriannual experience in cycling training and data analysis. When a data was incorrect due to technological issues (i.e. unusual high or low GPS, heart rate or power data) or incomplete due to faults in the start and stop of the registration, it was excluded. The dataset was exported to Microsoft Excel and explored for data spikes that were manually eliminated. The inclusion criterium was the presence of at least 20 files per month for the analyzed period considered. The percentage of complete data was 85%.

### Training Dose Indices: Training load and Training Intensity distribution

Training dose indices were monitored daily both during training sessions and races. Training load (TL) was measured using both heart rate and mechanical power, according to Edwards’ TRIMP (eTRIMP) and Training Stress Score, respectively.

eTRIMP [30] was calculated based on the time spent in five HR zones, multiplied by a zone-specific arbitrary weighting factor and then summated to provide a total TRIMP score: zone 1: 50–59% HRpeak, weighting factor = 1; zone 2: 60–69% HRpeak, weighting factor = 2; zone 3: 70–79% HRpeak; weighting factor = 3; zone 4: 80–89% HRpeak, weighting factor = 4; zone 5: 90–100%; weighting factor = 5. HRpeak was defined as the highest HR registered by the participant during training or race of the analyzed season.

TSS [31] was calculated using the following formula:

$$\begin{array}{c} Polarization\;Index\;(AU)=\\ =log10(Zone1/Zone2*Zone3*100) \end{array}$$

where t indicates the duration of the training session in seconds, NP™ the normalized power [31] and IF™ the ratio between NP™ and the functional threshold power (FTP). FTP was defined as the highest power output a cyclist can maintain in a quasi-steady state for approximately 60 min [32]; it was estimated by subtracting the five percent to the highest mean of twenty minutes power output recorded in race or training (RPO20) [33] and was updated on an annual basis.

Training intensity distribution was calculated using a three-zone power-based model: FTP was used to separate Zone 2 and Zone 3, while the 80% of the FTP was arbitrarily used to separate Zone 1 and Zone 2 because it represents an exercise intensity close to the first ventilatory threshold. The Polarization Index (PI) [34] was the second index used to monitor the training intensity distribution. PI has the advantage of summarizing in a single variable the entire intensity distribution. Essentially, as this number increases, TID polarization increases (higher percentages of time spent in both high- (Z3) and low-intensity exercise (Z1), compared to training time spent in medium intensity exercise (Z2). In the same way, if PI decreases, TID polarization decreases (i.e. higher percentages of time spent in Z2). In the training dose-performance relationships, eTRIMP, TSS, Z1, Z2, and Z3, were calculated as the sum of the daily values registered during the period taken into consideration. PI was calculated considering the sum of Z1, Z2 and Z3 for the period analyzed according to the following formula [34]:

$$TSS=\left[\left(t*NP^{™}*IF^{™})/(FTP*3600\right)\right]*100$$

where Zone is the fraction (given percentage/100) of the training volume in Zone 1, 2, and 3.

### Physical Performance: Record Power Outputs (RPOs)

RPOs over four different time durations calculated using a cycling performance software analyzer (Today’s Plan Pty Ltd, Australia) were taken into consideration: 1 min, 5 min, 20 min and 40 min (RPO1, RPO5, RPO20 and RPO40). As indicated by Pinot and Grappe [24] the term “record power output” is the highest power output produced by the cyclist and it is not his maximum achievable. Since in our database cyclists’ weight was not regularly updated, RPOs were calculated only in term of absolute (W) and not relative (W · Kg^.1^) power.

### Training Dose-Physical Performance relationship

The training dose variables were correlated to RPOs across four different time periods.

- TL and TID of the previous month with RPOs recorded during the subsequent month (Monthly analysis).
- TL and TID of the 8 weeks preceding all goal races with RPOs recorded during these races (All races analysis);
- TL and TID of the 8 weeks preceding grand tours (Giro d’Italia,Tour de France, Vuelta Espana) goal races with RPOs recorded during these races (Grand Tours analysis);
- TL and TID of the 8 weeks preceding one-day goal races with RPOs recorded during these races (One-day races analysis).

The goal races were selected with the help of the team’s head of performance who examined backwards the training programs of each cyclist.

### Statistical Analysis

All training dose and physical performance parameters for all the four different time periods are presented as mean ± standard deviation. After normality of data was checked, multilevel mixed-model analysis (R: A Language and environment for statistical computing, Vienna, Austria) was employed to explore the relationship between TL and TID measures (eTRIMP, TSS, Z1, Z2, Z3, PI) with performance measures (RPO1, RPO5, RPO20, RPO40) in all the four time frames (Monthly, All races, Grand Tours, and One-day goal races analyses). The training related measures were included in the mixed model as a fixed effect (the variable on which we want the inference being made), while individual cyclist was included as a random effect to take into account the dependency of the repeated measures. All models included random intercept and random slope terms to account for potential inter-individual variability in the baseline performance and different responsiveness to training load, respectively, as previously done in elite sport analyses [35]. Alpha level was set *a priori* at 0.05. Effect sizes were calculated using Cohen’s d [36]. Uncertainties in the correlation coefficients are presented as 95% confidence intervals and interpreted as trivial (0–0.09), small (0.1–0.29), moderate (0.3–0.49), large (0.50–0.69), very large (0.70–0.89), nearly perfect (0.90–0.99), perfect (1.00) [36].

From the statistical analysis emerged that p values of some small-to-moderate relationships were included between 0.05 and 0.1. For this reason, *a posteriori* an alpha level of 0.1 was considered to include among the Results p levels < 0.1 and discuss those relationships accordingly. It is acknowledged that for those relations the chance of occurring a Type I error was inflated to 10%.

## RESULTS

All the training dose and physical performance parameters for the four different time periods considered are presented in Table 1.

**TABLE 1:** Training dose and physical performance parameters for the four time periods considered: Monthly Analysis, All Races Analysis, Grand Tours Analysis and One-day Races Analysis. Data are presented as mean ± SD.

	Time periods	Monthly Analysis 4 weeks	All Races Analysis 8 weeks	Grand Tours Analysis 8 weeks	One-day Races Analysis 8 weeks
**Training Dose**	**Time (hr)**	78 ± 7	150 ± 16	151 ± 19	148 ± 21
	**eTRIMP (AU)**	8573 ± 1568	17041 ± 2959	17020 ± 3473	16657 ± 3664
	**TSS (AU)**	3290 ± 513	6536 ± 955	6481 ± 885	6547 ± 1245
	**Z1 (hr)**	54 ± 8	104 ± 15	106 ± 18	98 ± 16
	**Z2 (hr)**	12 ± 3	22 ± 6	22 ± 7	22 ± 7
	**Z3 (hr)**	6 ± 2	11 ± 4	11 ± 4	13 ± 5
	**Z1 (%)**	76 ± 5	76 ± 5	76 ± 5	74 ± 5
	**Z2 (%)**	16 ± 3	16 ± 3	16 ± 4	16 ± 4
	**Z3 (%)**	8 ± 2	8 ± 3	8 ± 3	9 ± 3
	**PI (AU)**	1.58 ± 0.12	1.58 ± 0.13	1.55 ± 0.13	1.61 ± 0.14

**Physical Performance**	**RPO1 (W)**	591 ± 61	585 ± 56	614 ± 73	575 ± 56
	**RPO5 (W)**	434 ± 33	435 ± 36	452 ± 35	427 ± 32
	**RPO20 (W)**	363 ± 28	399 ± 29	392 ± 26	350 ± 28
	**RPO40 (W)**	330 ± 25	366 ± 26	363 ± 22	324 ± 28

Abbreviations: TIME, total exercise time; eTRIMP, Edwards TRIMP; TSS, Training Stress Score; Z1: time spent in Zone 1; Z2: time spent in Zone 2; Z3: time spent in Zone 3; PI: polarization index; RPO1–RPO5–RPO20–RPO40: Record Power Output for 1–5– 20–40 minutes durations.

### Monthly analysis

**FIG. 1:**
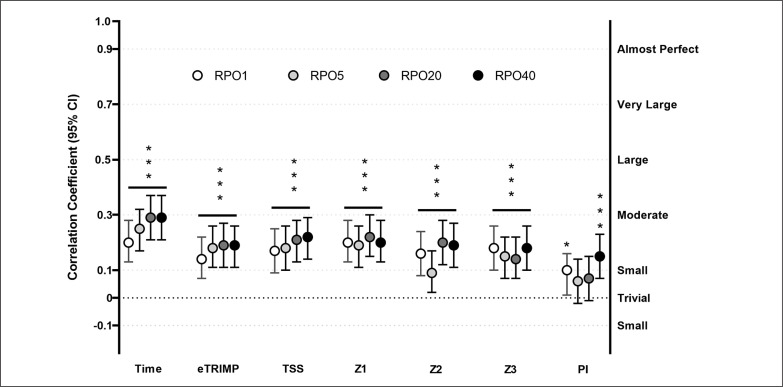
Relationship between monthly training load and training intensity distribution and Record Power Outputs for four different durations (1, 5, 20, and 40 min) registered in the subsequent month. Correlation coefficients (r) are presented with 95% confidence intervals (CI). Abbreviations: TIME, total exercise time; eTRIMP, Edwards TRIMP; TSS, Training Stress Score; Z1: hours spent in Zone 1; Z2: hours spent in Zone 2; Z3: hours spent in Zone 3; PI: polarization index; RPO1 – RPO5 – RPO20 – RPO40: Record Power Output for 1–5–20–40 minutes durations. Number of observations = 1278; Riders: 46; * = p < 0.05; ** = p < 0.01; *** = p < 0.001.

There were significant (p ≤ 0.001) small (r between 0.10 and 0.29, Fig 1) positive relationships between all the training load measures, except for PI, and all the four absolute RPOs (RPO1, RPO5, RPO20, RPO40). PI showed small significant positive associations with RPO1 (r = 0.10, p = 0.040) and RPO40 (r = 0.15, p ≤ 0.001).

### All races analysis

**FIG. 2:**
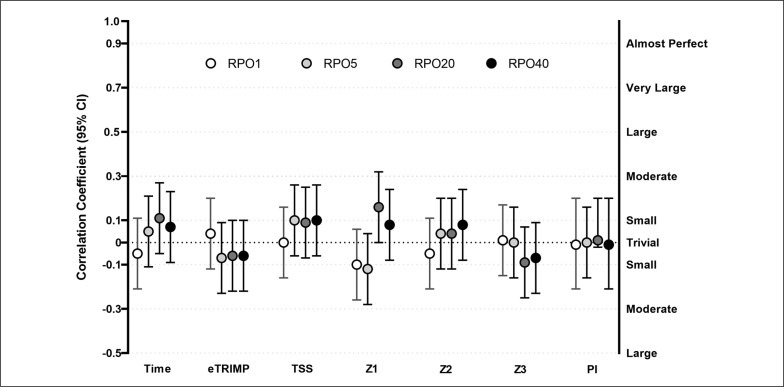
Relationship between training load and training intensity distribution of the 8 weeks preceding all goal races (A) and absolute Record Power Outputs registered during these races. Abbreviations: TIME, total exercise time; eTRIMP, Edwards TRIMP; TSS, Training Stress Score; Z1: hours spent in Zone 1; Z2: hours spent in Zone 2; Z3: hours spent in Zone 3; PI: polarization index; RPO1 – RPO5 – RPO20 – RPO40: Record Power Output for 1 – 5 – 20 – 40 minutes durations. Number of observations: 276; Riders: 30.

There were no significant relationships between any of the training load variables and RPOs (r between -0.12 and 0.16, all p > 0.079, Fig 2)

### Grand Tour analysis

**FIG. 3:**
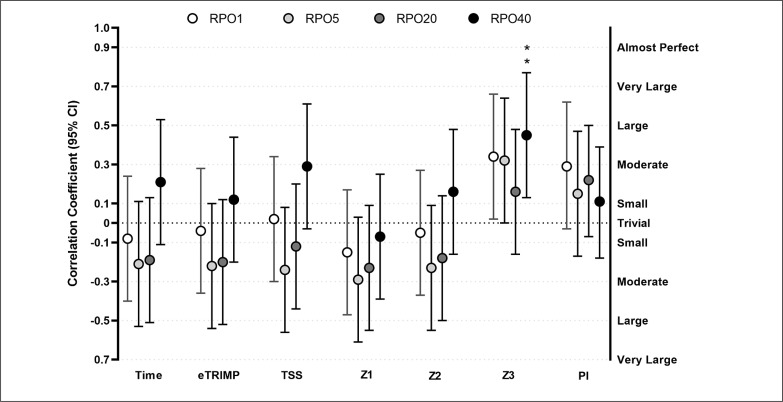
Relationship between training load and training intensity distribution of the 8 weeks preceding grand tours goal races and absolute Record Power Outputs registered during these races. Abbreviations: TIME, total exercise time; eTRIMP, Edwards TRIMP; TSS, Training Stress Score; Z1: hours spent in Zone 1; Z2: hours spent in Zone 2; Z3: hours spent in Zone 3; PI: polarization index; RPO1 – RPO5 – RPO20 – RPO40: Record Power Output for 1–5–20–40 minutes durations. Number of observations: 74; Riders: 21; ** = p < 0.01.

Z3 showed a moderate positive associations with RPO40 (r = 0.45, p = 0.007). With alpha set at 0.1, Z3 was positively related to RPO1 and RPO5 (r between 0.32 and 0.34, p = 0.053–0.059, moderate). Similarly (alpha at 0.1), PI was positively related to RPO1 (r = 0.29, p = 0.076, small,). There were no relationships between any of the others training indices and RPOs (Fig. 3).

### One-day goal races analysis

**FIG. 4:**
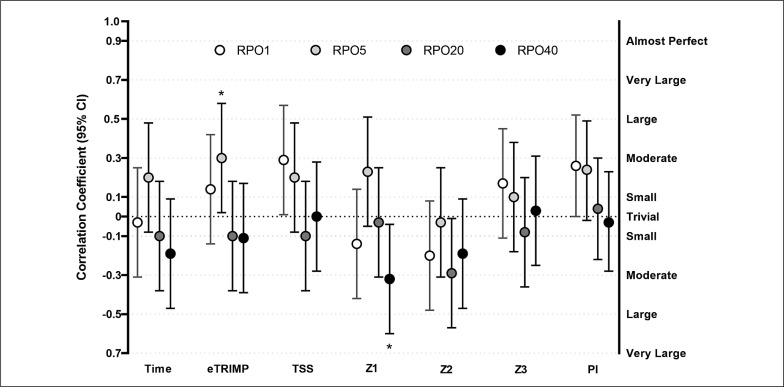
Relationship between training load and training intensity distribution of the 8 weeks preceding one-day goal races and absolute Record Power Outputs registered during these races. Abbreviations: TIME, total exercise time; eTRIMP, Edwards TRIMP; TSS, Training Stress Score; Z1: hours spent in Zone 1; Z2: hours spent in Zone 2; Z3: hours spent in Zone 3; PI: polarization index; RPO1 – RPO5 – RPO20 – RPO40: Record Power Output for 1–5–20–40 minutes durations. Number of observations: 94. Riders: 18; * = p < 0.05.

eTRIMP was positively related to RPO5 (r = 0.30, p = 0.035, moderate). Z1 was negatively related to RPO40 (r = -0.31, p = 0.031, moderate). When alpha was set at 0.1, PI was positively related to RPO5 (r = 0.24, p = 0.068, small) and Z2 negatively related to RPO20 (r = – 0.29, p = 0.051, small). There were no relationships between any of the others training indices and RPOs (Fig. 4).

## DISCUSSION

The main findings of the present study highlighted that higher monthly training dose indices were associated with higher indices of physical performance (i.e. RPOs) recorded in the next month, however the magnitude of the relationships found were small. Only trends and a few significant small-positive relationships were found between some training dose indices and some indices of physical performance achieved during all races, Grand Tours, and one-day goal races. Descriptive data seems to be in accordance with previous studies on professional road cyclists regarding both training exposure (~900–950 hours per year) [27] and RPOs for the difference time durations [24]. In a recent interesting study, Emig and Peltonen [37] performed a big data analysis (≈14,000 individuals) correlating training dose and performance in runners of different performance levels. Interestingly, they observed an initial linear increase of performance with TRIMP, a plateau around a certain value of TRIMP, and then a statistically significant drop which may be due to overtraining. While the cited study comprehended a wide range of training loads and performance levels, we considered only a narrow selection of performance level, which corresponds to the highest one (i.e. professional level). Hence, it might be that our study considered only the plateau section of Emig and Peltonen’s relationship, which represents professional athletes working at very high workloads, near the highest point of functional over-reaching [38] just before the threshold of overtraining, [39] and already possessing very high level of physical performance. This might be the reason why we observed only trends or small relationships between training dose and performance. In addition, the very large difference in the numerosity of the samples between the two studies can be a further reason that can explain the diversities of the main results.

Our findings are different from previous studies investigating the relationship between training load and performance in competitive cyclists. Sanders and Colleagues [3] found large to very large relationships between training load and performance. This could be due to the different performance measures utilized that have both pros and cons to bear in mind: a 8-min time trial test, in the study by Sanders et al. [3], which is standardized but less ecological, and field RPOs in the present study which are ecological but less standardized and need to be critically evaluated regarding validity and reliability [40]. Furthermore, in the present study, data from *All races*, *Grand Tour, One-day goal races analyses* were all collected during the competitive periods of the seasons, while Sanders and colleagues [3] studied only the pre-season training period. This latter typically consists in low-intensity high-volume training and usually changes in performance are greater in this period compared to those achieved in all the other periods of the seasons [41]. On the other hand, the competitive period of professional road cyclists typically involves more time spent at high-intensity and more periods of high physical and psychological stress [42]. It is therefore reasonably fair assuming that the detection of relationships between changes in performance and training dose is easier in the first scenario proposed by Sanders et al. [3] than in ours. Accordingly, Leo and Colleagues [28], in the other study correlating training dose and performance on competitive cyclists, reported as changes in training characteristics correlated with changes in the power profile in early- and mid-season, but not in late-season in U23 competitive cyclists.

### Monthly analysis

Differently from other analyses, the monthly analysis highlighted a significant positive effect of all training dose variables on RPOs. This may be due to the much higher number of observations included in the Monthly analysis (n = 1278) compared to All races (n = 276), Grand tour (n = 74) and One day (n = 94) goal races analyses, which could have facilitated the achievement of statistical significance. In addition, professional road cyclists’ training workload presents within season fluctuations according to the different macrocycles (e.g. general base preparation, racing, event preparation) [43]. In this perspective, while the three goal races analysis included only “racing” and “event preparation” macrocycles, Monthly analysis incorporated also the “general base preparation” macrocycle and thus probably included more workload fluctuations compared to other analysis, increasing the chances to observe significant relationships between training dose and physical performance parameters. The mixed modelling approach used in the present study may be considered an appropriate tool to detect this periodical fluctuation during the seasons within and between individuals [44].

It should be bore in mind that elite cyclists, such as those considered in the present study, are usually subjected to very high training loads all the yearlong and for many years [45]. This issue might influence the detection of a relationship between training dose and physical performance as they may already have reached the plateau of their maximum physical performance potential. Despite this, the significant positive effects of training dose on absolute RPOs could suggest that a certain degree of responsiveness to training dose persists even in professional endurance athletes.

### All races, Grand Tour, One-day goal races analyses

In Grand Tour analysis the moderate positive relations of RPO40 to Z3 and trends to moderate positive relations of RPO1-5 to Z3, could suggest to foster high-intensity (Z3) training during the preparation of these competitions. This may happen because excessively extensive loads may lead to a reduction in the activity of the pineal gland, adrenal glands, and testis which are already under great stress in these kind of competitions [46], where cyclists perform twenty-one consecutive days of race with only 2–3 days of rest. However, this should be contextualized within the very high exercise volume (about 22 hours a week) performed by the professional road cyclists analyzed in this study, and this should not be misunderstood as a recommendation to perform low volume training programs in the eight weeks preceding cycling grand tours. In One-day races analysis, the moderate positive relations of eTRIMP with RPO5, of Z1 with RPO40 and of PI – with RPO1, and the tendency of Z2 to be small negatively related to RPO20, could suggest to foster high complessive training load (eTRIMP) with a more polarized intensity distribution in preparation of these competitions. This reflects the physiological model of these races, which are long races not including long climbs with the final result often decided through explosive short duration efforts (i.e < 5′). Thus, those races could require both a high level of stamina and anaerobic qualities, which may require high training load with a certain amount of high intensity training (high eTRIMP with a more polarized intensity distribution).

**FIG. 5:**
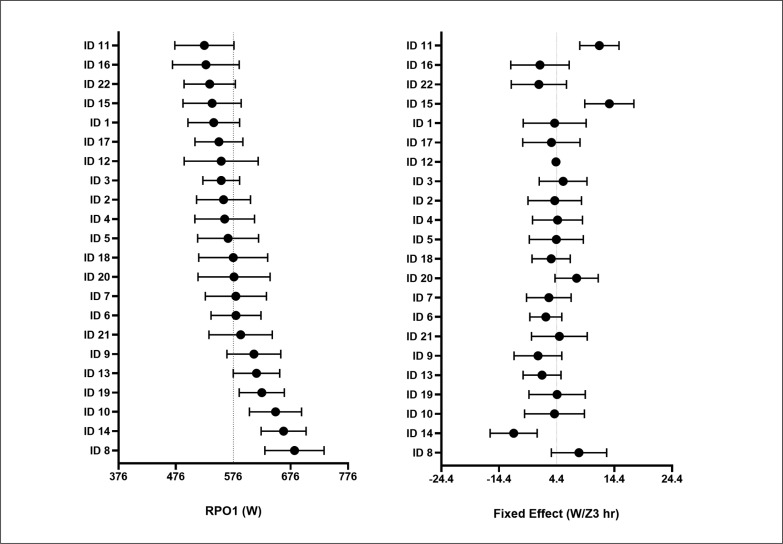
Individual variations in RPO1 for Grand Tours analysis following an increase of one hour spent in Z3 (“fixed effect”) during the eight weeks preceding grand tours. Each subject’s fixed effect is represented as mean (black circles) and standard deviation(lines). Abbreviations: Z3: hours spent in Zone 3; RPO1: Record Power Output for 1-minute duration.

However, all these considerations might be seen more as speculations instead of factors supported by strong evidence, as they are derived from the observation of trends and/or relationships with very large confidence intervals. A qualitative and quantitative representative example of all observed significant relationships and trends (Fig. 5) shows a very high inter- and intraindividual variability. Figure 5 represents an example of each cyclist’s variations in RPO1 in grand tours following an increase of one hour spent in Z3 (the so-called: “fixed effect”) in the previous 8 weeks. With regards to inter-individual variability, it can be observed that, although the average results highlighted a tendency to a positive effect of Z3 on RPO1, different cyclists showed different average behaviors with someone even recording a negative average fixed effect (i.e., they generally worsen their RPO1 after an increase of Z3). For what concerns intraindividual variability, it can be observed that each cyclist’s fixed effect confidence intervals are greater than the mean fixed effect itself, ranging from positive to even negative values. This suggests that at individual level an increase of training load could lead both to improve and to worsen RPOs, both in different cyclists and even within the same cyclist. This might suggest that professional cyclists are already exposed to high average level of training loads, and thus a further increase in physical stress could lead in some cases to non-functional overreaching and a consequent decrease in performance [39].

Among the factors that may have contributed to the high variability of some results, there is certainly the issue that RPOs can be influenced by other factors not included in the analysis, such as the category of the race [25] (Monuments, World Tour, Hors Catégorie, category 1) and the race type (flat, semi-mountainous, mountainous) [14]. In addition, in the present study, psychological factors were not considered and analyzed but it must be acknowledged that they can represent an important stimulus for the achievement of RPOs.

### Limitations and future directions

Important limitations of the present study, that might represent threats to its validity, must be acknowledged. First, a retrospective analysis was performed on a database not collected and daily checked for scientific purposes, consequently it had some missing data we tried to solve using narrow inclusion criteria. In addition, in Grand tours and One-day races analyses, a low number of observations were recorded for some subjects. Regarding this issue, possible solutions for future studies could be a daily controlled data collection and an increase in the number of data available by extending the data collection over multiple years or by including more than one team like in another research [37].

From a conceptual point of view, the use of RPOs as a performance measure remains questionable. In fact, RPOs could be daily influenced by factors such as: motivation, tactical factors, role of the cyclist, category of the race, and race type. This may have mixed up the results in All-races and One-day races analyses. One of the possibilities to overcome this issue is to introduce some of these factors influencing RPOs as further covariates in the model: the role of the cyclist (leader, domestique, etc….), category of race (Monuments, World Tour, Hors Catégorie, category 1), race type (flat race, semi mountainous stage, mountainous stage). In addition, in Grand tours, the ability to repeat high-level performances for consecutive days could be a more valid performance index than considering one isolated best performance such as RPOs. Furthermore, RPOs does not consider repeated efforts over time which could also be crucial in deciding race results. Regarding this, introducing a new power meters derived performance parameter, ordering power files in descending order and then calculating the average of the first hour, can be one of the possible solutions. It is suggested that future studies look also at different methods to express training intensity distribution [43], other than those used in the present study, to describe their relationships with performance indices.

Unfortunately session-RPE [47] was not included within the training dose parameters as it was not systematically collected by the team staff. Session-RPE is an internal load measure considering not only physiological factors but also the psychological ones [48] and it has been shown to be more sensitive to accumulated fatigue than HR [49]. Since the neurobiological context, in turn, greatly influenced by the background levels of psycho-emotional stress, influences fitness adaptations subsequent to imposed training stressors [50], session-RPE could be a more sensitive workload measure compared to HR- and power-based load parameters when investigating the relationship between training dose and performance.

Lastly, strength training in the gym was not included in training dose as it was not systematically recorded. Since it has been shown as strength training induces functional adaptations promoting cycling performance [51] future studies will also have to consider gym’s strength training load when investigating the relationship between training dose and performance in road cyclists.

## CONCLUSIONS

This explorative study is the first that tries to understand whether a clear relationship exists between training load and performance in professional road cyclists across all season’s periods.

Monthly analysis highlighted a significant positive effect of all training dose variables on RPOs suggesting that a certain degree of responsiveness to training dose persists even in professional endurance athletes.

Mixed results with very high levels of intra- and inter-individual variability were found in the analysis on goal races (all races, grand tours, one-day) with trends to significance which may suggest: (i) increase high intensity training (Z3) in preparation of grand tours; (ii) fostering high intensity training (Z3) and high complessive training load (eTRIMP and TSS) with a more polarized distribution in preparation of one-day races.
